# Inhibition of the PERK/TXNIP/NLRP3 Axis by Baicalin Reduces NLRP3 Inflammasome-Mediated Pyroptosis in Macrophages Infected with *Mycobacterium tuberculosis*

**DOI:** 10.1155/2021/1805147

**Published:** 2021-11-08

**Authors:** Yan Fu, Jingjing Shen, Yinhong Li, Fanglin Liu, Bangzuo Ning, Yuejuan Zheng, Xin Jiang

**Affiliations:** Center for Traditional Chinese Medicine and Immunology Research, School of Basic Medical Sciences, Shanghai University of Traditional Chinese Medicine, Shanghai 201203, China

## Abstract

*Mycobacterium tuberculosis* (Mtb) remains a significant threat to global health as it induces granuloma and systemic inflammatory responses during active tuberculosis. Mtb can induce macrophage pyroptosis, leading to the release of IL-1*β* and tissue damage, promoting its spread. Here, we established an in vitro Mtb-infected macrophage model to seek an effective antipyroptosis agent. Baicalin, isolated from Radix Scutellariae, was found to reduce pyroptosis in Mtb-infected macrophages. Baicalin could inhibit activation of the PERK/eIF2*α* pathway and thus downregulates TXNIP expression and subsequently reduces activation of the NLRP3 inflammasome, resulting in reduced pyroptosis in Mtb-infected macrophages. In conclusion, baicalin reduced pyroptosis by inhibiting the PERK/TXNIP/NLRP3 axis and might thus be a new adjuvant host-directed therapy (HDT) drug.

## 1. Introduction

As one of the top 10 causes of death worldwide and the leading cause of death caused by a single infectious agent, tuberculosis (TB) is a communicable disease caused by *Mycobacterium tuberculosis* (Mtb). Generally, Mtb can induce excessive inflammatory responses by infecting macrophages in the host, exacerbating tissue damage and patient symptoms [1]. Over the past two decades, research on TB and the development of drugs against TB have been a concern. However, owing to antimicrobial resistance and a lack of targeting drugs, these methods have not been satisfactory [2]. Therefore, a new approach to treat TB is urgently needed.

Host-directed therapy (HDT) is an emerging treatment concept that utilizes drugs to enhance protective immune responses against the pathogen or reduce exacerbated inflammatory responses, to finally balance immune reactivity and protect the host [3]. Since the excessive inflammatory response of macrophages induced by Mtb infection is the key factor in the pathological damage to a TB patient, the most important measure to alleviate the symptoms of patients is suppressing the excessive inflammatory response when the bacteria cannot be cleared in time [4]. Thus, finding drugs based on HDT might help the host immunity system “correctly” respond to Mtb infection and indirectly enhance the effectiveness of anti-TB drugs.

Baicalin is a constituent of Radix Scutellariae and exerts strong biological effects, such as anti-inflammatory [5], blood pressure-reducing [6], and glucose and lipid metabolism regulatory activities [7]. Moreover, baicalin can attenuate H_2_O_2_-induced cytotoxicity by reducing ER stress and activating Nrf2 signaling [8]. Our previous study demonstrated that baicalin can suppress inflammatory responses in Mtb-infected macrophages by inducing autophagy through the PI3K/Akt/NF-*κ*B signaling pathway [9]. Here, we found a new mechanism through which baicalin alleviates TB, in which it could inhibit pyroptosis in Mtb-infected macrophages to reduce excessive inflammatory responses.

## 2. Material and Methods

### 2.1. Chemicals and Reagents

RIPA lysis buffer, BCA protein assay kit, Protein A/G agarose/sepharose beads, and Lactate Dehydrogenase (LDH) Cytotoxicity Assay Kit were purchased from the Beyotime Institute of Biotechnology (Shanghai, China). The following antibodies were used: anti-BIP (3177S), anti-CHOP (2895T), anti-NLRP3 (15101S), IRE1a (3294T), and p-eIF2*α* (3398T) were purchased from Cell Signaling Technology, Inc.; anti-GSDMD (A18281) was obtained from ABclonal Technology (Wuhan, China); anti-TXNIP (sc-166234) was bought from Santa Cruz Biotechnology, Inc.; anti-*β*-actin monoclonal antibody was purchased from Proteintech Group (Chicago, IL); goat anti-NLRP3 (49012) was purchased from SAB Laboratories, Inc. (Logan, UT, USA). Middlebrook 7H9 and 7H10 media were obtained from Difco (Detroit, MI, USA), and oleic acid-albumin-dextrose-catalase (OADC) supplements were from BD Biosciences (BD, Sparks, MD, USA). RiboFECT CP Transfection Kit was purchased from Ribo Biotechnology (Guangzhou, China). IL-1*β* ELISA Kit was purchased from R&D (USA).

### 2.2. Animals

Male C57BL/6J mice (6-8 weeks, 20 ± 3 g) were obtained from Vital River Laboratory Animal Technology Co., Ltd. (Beijing, China). All mice were acclimated for at least 1 week before the experiments and housed in a pathogen-free facility under standard condition (4 per cage, 12-hour-light/dark cycle, lights on from 7:00 AM to 7:00 PM; 22°C ± 1°C ambient temperature; 56% ± 10% relative humidity; noise < 50 dB; ammonia concentration < 14 mg/m^3^; bedding replacement once a week), with free access to food and water. Intraperitoneal injection of 2 ml of 3% sodium thioglycolate and the primary peritoneal macrophage cells were collected after three days. For animal sacrifice, all mice were anaesthetized using isoflurane and then killed by cervical dislocation. Animal experiments strictly follow the National Institutes of Health Guide for the Care and Use of Laboratory Animals, with the approval of the Scientific Investigation Board of Shanghai University of Traditional Chinese Medicine (Shanghai, China).

### 2.3. Drugs

Baicalin (molecular weight: 446.37, purity > 98%, the chemical structure shown in Fig. S1) is extracted from the Radix Scutellariae (the full names have been checked according to http://www.theplantlist.org). Baicalin was purchased from Shanghai Tauto Biotech Co., LTD. (CAS: 21967-41-9) (Shanghai, China). Firstly, it was dissolved with DMSO to a storage concentration of 100 mM and stored at -20°C, and then, it was diluted to 100 *μ*M with DMEM containing 10% FBS for the following experiments.

### 2.4. Cell Culture

Mouse macrophage-like cell line Raw264.7 was purchased from ATCC (Manassas, VA) and cultured in DMEM supplemented with 10% fetal bovine serum (FBS) in 5% CO_2_ at 37°C. Thioglycolate-elicited mouse primary peritoneal macrophages were prepared from male C57BL/6J mice (6-8 weeks of age) as described previously [10]. After 2 h, nonadherent cells were removed and the adherent cells were used as primary peritoneal macrophages.

### 2.5. Bacterial Strains

The Mtb H37Ra was used in this study. H37Ra strain was grown in Middlebrook 7H9 or 7H10 broth supplemented with 0.2% glycerol, 0.05% Tween-80, and 10% Middlebrook OADC supplement.

### 2.6. Mtb Infection

The Raw264.7 cells or primary peritoneal macrophage cells were seeded at various specifications of the cell culture plates and grown at 37°C overnight. The cells (the density of Raw264.7 cells was 3 × 10^6^, 4 × 10^5^, or 8 × 10^5^; the density of primary peritoneal macrophage cells was 6 × 10^6^, 8 × 10^5^, or 1.5 × 10^6^) were infected with Mtb H37Ra at a MOI (multiplicity of infection) of 10 : 1. After 4 h of coincubation at 37°C, cells were washed three times with sterile phosphate-buffered saline (PBS) and cultured with DMEM containing 10% FBS in the presence and absence of baicalin (100 *μ*M) for different times (6, 12, and 24 hours). The experimental groups are control, Mtb 6 h, Mtb+baicalin 6 h, Mtb 12 h, Mtb+baicalin 12 h, Mtb 24 h, and Mtb+baicalin 24 h.

### 2.7. Western Blotting

The Raw264.7 cells or primary peritoneal macrophage cells were seeded at 6-well cell culture plates (Raw264.7 cells: 8 × 10^5^ cells/well; primary peritoneal macrophage cells: 1.5 × 10^6^ cells/well) and grown at 37°C overnight. The cells were infected with Mtb H37Ra (MOI = 10 : 1). Then, cells were washed three times with sterile PBS and cultured with DMEM containing 10% FBS in the presence and absence of baicalin (100 *μ*M) for different times (6, 12, and 24 hours). Cells were collected and lysed in RIPA lysis buffer (Beyotime Institute of Biotechnology, China) after different times, and then, the total protein concentration of each sample was detected by a BCA protein assay kit. Then, the whole cell lysate was added 5x SDS and boiled 10 minutes in 100°C and separated by SDS-PAGE and transferred onto nitrocellulose membranes. The membranes were incubated with different primary antibodies at 4°C overnight after blocking with 5% (*w*/*v*) bovine serum albumin dissolved in TBST (1% Tween-20); the dilution ratio of each primary antibody was 1 : 1000. The membranes were incubated with HRP-conjugated secondary antibodies (1 : 10000) at room temperature for 2 hours after being washed by TBST three times. Chemiluminescence was tested with ECL-chemiluminescent kit (Thermo Scientific). The quantification of each protein was performed by ImageJ (1.51 (100), 2015).

### 2.8. Coimmunoprecipitation

The Raw264.7 cells or primary peritoneal macrophage cells were seeded at 30 mm dish culture plates (Raw264.7 cells: 3 × 10^6^ cells/well; primary peritoneal macrophage cells: 6 × 10^6^ cells/well) with three groups (control, Mtb 12 h, and Mtb+baicalin 12 h). The cells were infected with Mtb H37Ra (MOI = 10 : 1). Then, cells were washed three times with sterile PBS after 4 hours and cultured with DMEM containing 10% FBS in the presence and absence of baicalin (100 *μ*M) for 12 hours. Raw264.7 cells or primary peritoneal macrophages were lysed by ice-cold cell lysis buffer, and cell lysates were cleared by centrifugation (12000 rpm, 10 min). Before immunoprecipitation, samples containing equal amounts of protein were precleared with specific antibodies (1-2 mg/ml) overnight at 4°C with gentle rotation and then incubated with Protein A/G agarose/sepharose beads at 4°C with gentle rotation for four hours. Then, agarose/sepharose beads were washed extensively with lysis buffer four times, and proteins were eluted by boiling in 1x SDS buffer before SDS-PAGE electrophoresis. The steps after SDS-PAGE electrophoresis are the same as western blotting measurement.

### 2.9. Immunofluorescence

The Raw264.7 cells were seeded at culture plates (7 × 10^4^ cells/well) with three groups (control, Mtb 12 h, and Mtb+baicalin 12 h) and grown at 37°C overnight. The cells were infected with Mtb H37Ra (MOI = 10 : 1). Then, cells were washed three times with sterile PBS after 4 hours and cultured with DMEM containing 10% FBS in the presence and absence of baicalin (100 *μ*M) for 12 hours. The cells were washed three times with 37°C prewarmed PBS and fixed with 37°C prewarmed 4% paraformaldehyde at room temperature for 15 min, and then, the cells were washed three times with 37°C prewarmed PBS again. The cells were treated with penetrating reagents (0.2% of BAS, 2% of Triton X-100) for 15 min at room temperature and washed three times with 37°C prewarmed PBS, and then, the cells were blocked with 5% bovine serum albumin for 0.5 hours at room temperature. Rabbit anti-NLRP3 and mouse anti-TXNIP antibodies were used for immunofluorescence, primary antibodies were dissolved in 5% bovine serum albumin, and the dilution ratio of each primary antibody was 1 : 200. The cells were washed three times with 37°C prewarmed PBS after being incubated with primary antibodies at 4°C overnight. Donkey anti-mouse IgG and goat anti-rabbit IgG antibodies were used as secondary antibodies; the dilution ratio of each secondary antibody was 1 : 200. The cells were incubated with secondary antibodies for 2 hours. Then, the cells were washed three times with 37°C prewarmed PBS. The nuclei were stained with DAPI at the concentration of 2 *μ*g/ml for 10 min. Then, the cells were washed three times with 37°C prewarmed PBS. All operations should be protected from light from the step of adding the secondary antibodies. Confocal microscopy (LSM 880, Zeiss Optics International Trading Co., LTD.) was used for examination. The quantification of each protein was performed by ImageJ (1.51 (100), 2015).

### 2.10. IL-1*β* ELISA

The Raw264.7 cells were seeded at 24-well plates (2 × 10^5^ cells/well) with three groups (control, Mtb 12 h, and Mtb+baicalin 12 h) and grown at 37°C overnight. The cells were infected with Mtb H37Ra (MOI = 10 : 1). The cells were washed three times with sterilized PBS 4 hours later and then were added with or without baicalin (100 *μ*M) medium continually cultured for 24 hours. To measure the production of IL-1*β*, the supernatants of the cells were collected and measured using enzyme-linked immunosorbent assay (ELISA) kits purchased from R&D according to the manufacturer's protocol.

### 2.11. LDH Release Assay

Cell death was evaluated applying the Lactate Dehydrogenase (LDH) Cytotoxicity Assay Kit. The Raw264.7 cells or primary peritoneal macrophage cells were seeded at 24-well plates (2 × 10^5^ cells/well) with fifteen groups (control 6 h, Mtb 6 h, Mtb+baicalin 6 h, control 12 h, Mtb 12 h, Mtb+baicalin 12 h, control 24 h, Mtb 24 h, Mtb+baicalin 24 h, control 48 h, Mtb 48 h, Mtb+baicalin 48 h, control 72 h, Mtb 72 h, and Mtb+baicalin 72 h) and grown at 37°C overnight. The cells were infected with Mtb H37Ra (MOI = 10 : 1). The cells were washed three times with sterilized PBS 4 hours later and then were added with or without baicalin (100 *μ*M) medium continually cultured. After baicalin (100 *μ*M) treatment for 6, 12, 24, 48, and 72 hours, the chromogenic reagent provided in the LDH assay kit was then added, and the luminescence signals were measured using a microplate spectrophotometer. The relative amount of LDH was calculated according to the following formula: LDH relative release amount (%) = (absorbance of treated sample − absorbance of control hole of sample)/(absorbance of maximum enzyme activity of cells − absorbance of control hole of sample) × 100.

### 2.12. siRNA Transfection

RAW264.7 cells were seeded at six-well tissue culture plates at a density of 3 × 10^5^ cells per well with 2 ml of antibiotic-free DMEM (10% FBS) medium with six groups (control, Mtb, Mtb+siIRE1*α*, Mtb+siPERK, Mtb+siATF6, and Mtb+NC). The cells were incubated at 37°C in a CO_2_ incubator until about 80% of cells were confluent. This took approximately 24 hours. The following solutions were prepared: 1.858 ml antibiotic-free DMEM (10% FBS) medium, 120 *μ*l buffer, 12 *μ*l reagent, and 10 *μ*l siRNA (20 *μ*M). The solution was mixed gently by pipetting and incubated for 0-15 min at room temperature. The mixture was added to the 6-well plate. The cells were incubated for 48 hours. Then, the transfection efficiency was evaluated by western blotting to detect the IRE1*α*, p-eIF2*α*, ATF6, and GSDMD protein expressions. Higher interference efficiency siRNAs were selected for following researches, and Mtb was added after being transfected for 48 hours (MOI = 10 : 1). Cells were washed three times with sterilized PBS 4 hours later and then were added with or without baicalin (100 *μ*M) medium continually cultured for 12 hours. The cells were harvested and dissociated in ice-cold cell lysis buffer; subsequently, the proteins of whole cell were extracted and the target proteins are tested by western blotting eventually. The siRNA sequences are as follows: IRE1*α*-mus-963 (KD1) sense 5′-GCUAACGCCUACUCUGUAUTT-3′, antisense 5′-AUACAGAGUAGGCGUUAGCTT-3′; IRE1*α*-mus-1463 (KD2) sense 5′-CCAUUAU CCUGAGCACCUUTT-3′, antisense 5′-AAGGUGCUCAGGAUAAUGGTT-3′; IRE1*α*-mus-2523 (KD3) sense 5′-GGACGUCAUUGCUCGUGAATT-3′, antisense 5′-UUCACGAGCAAUGACGUCCTT-3′; EIF2AK3-mus-525 (PERK-KD1) sense 5′-GCACUUUAGAUGGACGAAUTT-3′, antisense 5′-AUUCGUCCAUCUAAAGUGCTT-3′; EIF2AK3-mus-1779 (PERK-KD2) sense 5′-GGACGAUCCUGCUUUGCAUTT-3′, antisense 5′-AUGCAAAGCAGGAUCGUCCTT-3′; EIF2AK3-mus-2683 (PERK-KD3) sense 5′-GCAUGAUGGCAACCAUUAUTT-3′, antisense 5′-AUAAUGGUUGCCAUCAUGCTT-3′; ATF6-mus-172 (KD1) sense 5′-GCACUUUGAUGCAGCACAUTT-3′, antisense 5′-AUGUGCUGCAUCAAAGUGCTT-3′; ATF6-mus-682 (KD2) sense 5′-GCAGUCGAUUAUCAGCAUATT-3′, antisense 5′-UAUGCUGAUAAUCGACUGCTT-3′; ATF6-mus-242 (KD3) sense 5′-CCUUGGGAGUCAGACCUAUTT-3′, antisense 5′-AUAGGUCUGACUCCCAAGGTT-3′; GSDMD-Mus-964 (KD1) sense 5′-CCUGUCAGAUGGGAUUGAUTT-3′, antisense 5′-AUCAAUCCCAUCUGACAGGTT-3′; GSDMD-Mus-1471 (KD2) sense 5′-GGUCUUGCUAGAAGAAUGUTT-3′, antisense 5′-ACAUUCUUCUAGCAAGACCTT-3′; GSDMD-Mus-495 (KD3) sense 5′-CCUCCAUGAAUGUGUGUAUTT-3′, antisense 5′-AUACACACAUUCAUGGAGGTT-3′; and negative control sense 5′-UUCUCCGAACGUGUCACGUTT-3′, antisense 5′-ACGUGACACGUUCGUAGAATT-3′.

### 2.13. Statistical Analysis

Statistical analysis was performed by using GraphPad Prism 8 (GraphPad Software, La Jolla, CA, USA). Statistical significance was determined using one-way ANOVA, and results were given as the means ± standard deviation (SD). Data shown are representative of at least triplicate experiments. A value of *p* < 0.05 was considered to be statistically significant.

## 3. Results

### 3.1. Baicalin Ameliorates Pyroptosis in Mtb-Infected Macrophages

Pyroptosis is a form of programmed cell death characterized by upregulated expression of caspase-1-cleaved gasdermin D (GSDMD), which forms membrane pores and promotes the release of proinflammatory mediators, including IL-1*β*, in response to the adverse effects of certain bacteria [11]. In TB patients, Mtb can induce macrophage pyroptosis. During this progress, the nucleotide-binding domain leucine-rich repeat and pyrin domain-containing receptor 3 (NLRP3) inflammasome is activated, leading to the release of interleukin-1*β* (IL-1*β*) and HMGB1 and promoting the spread of Mtb to neighboring cells [11–13].

**Figure 1:**
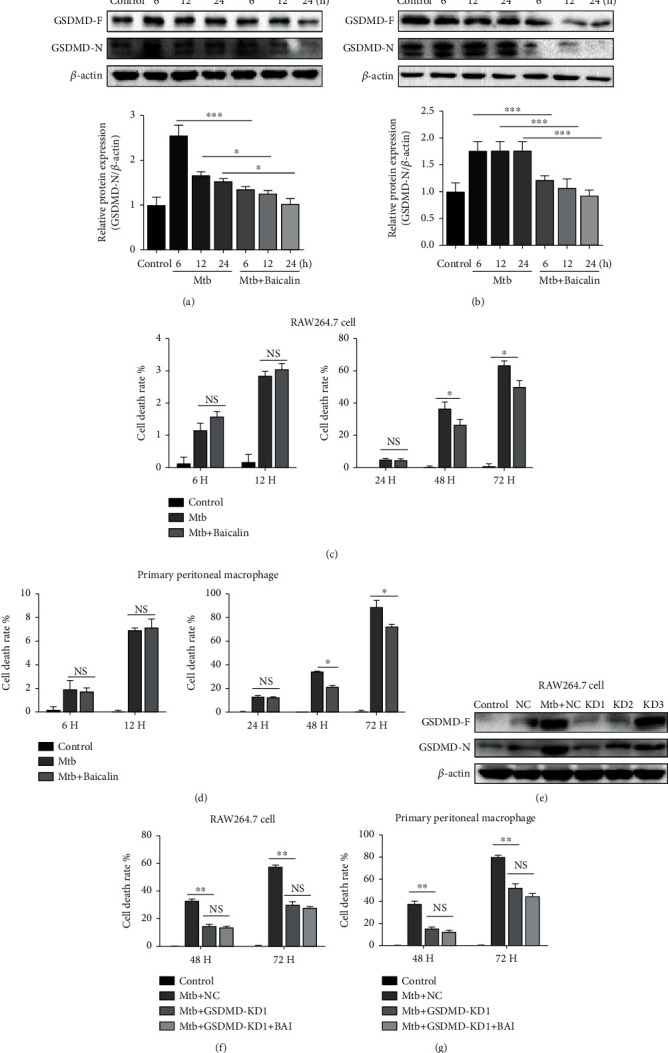
Baicalin ameliorates pyroptosis in Mtb-infected macrophages. (a) Levels of GSDMD-N protein were determined by western blotting in RAW264.7 cells. (b) Levels of GSDMD-N proteins were analyzed by western blot measurement in primary peritoneal macrophage cells. (c, d) Cell death was evaluated applying the Lactate Dehydrogenase (LDH) Cytotoxicity Assay Kit in RAW264.7 cells and in primary peritoneal macrophage cells. (e) Transfection efficiency of siRNAs was measured by western blotting analysis in RAW264.7 cells. (f, g) Cell death after GSDMD siRNA was evaluated applying the Lactate Dehydrogenase (LDH) Cytotoxicity Assay Kit in RAW264.7 cells and in primary peritoneal macrophage cells. Data are shown as mean ± SD of three independent experiments. ^∗^*p* < 0.05; ^∗∗∗^*p* < 0.001; NS: nonsignificant.

Our previous results suggested that baicalin can significantly inhibit NLRP3 inflammasome activation [9]. Thus, we determined whether baicalin could affect pyroptosis through the NLRP3 inflammasome. According to a previous MTT experiment, we used 100 *μ*M baicalin to treat Mtb-infected macrophages and then investigated GSDMD expression by western blotting. As shown in Figure 1, GSDMD-N protein levels were increased in RAW264.7 cells (Figure 1(a)) and primary peritoneal macrophages (Figure 1(b)) infected with Mtb, whereas treatment with baicalin significantly reduced GSDMD-N protein levels.

Moreover, lactate dehydrogenase (LDH) is a relatively more stable enzyme that is also released during pyroptosis. Thus, we detected LDH activity to assess the level of pyroptosis. The results revealed that Mtb infection could increase the release of LDH at different time points, whereas treatment with baicalin can reverse this effect within 48 h and 72 h in RAW264.7 cells and primary peritoneal macrophages (Figures 1(c) and 1(d)). Since LDH release not only occurs in pyroptosis but also occurs in other forms of cell death, the LDH release was assessed after GSDMD siRNA transfection. The results showed that LDH release was significantly reduced in Mtb-infected macrophages after GSDMD siRNA transfection, and there is no significance between the Mtb-infected group and the treatment of baicalin (Figures 1(e)–1(g)). This result demonstrates that LDH release in Mtb-infected macrophages partly depends on the upregulation of GSDMD-N. Moreover, our previous work also revealed that baicalin could reduce the release of IL-1*β* [9]. In addition, the level of HMGB1 in the supernatants was assessed. The results showed that baicalin treatment could reduce the expression of HMGB1 in 24 hours (Figure S2). Propidium iodide (PI), whose uptake indicates cell damage or death, was widely used to assess the viability of cells in different experimental models. To analyze whether baicalin affects the viability of macrophages, we determined the viability of macrophages in different groups by PI staining flow cytometric assay. The results of PI staining flow cytometric assay demonstrated that Mtb infection can increase the death or damage rates of macrophages at different time points both in RAW264.7 cells and in primary peritoneal macrophages, while treatment with baicalin can significantly abolish this effect within 24 h (Figure S3). These results indicated that baicalin can reduce pyroptosis by inhibiting NLRP3 inflammasome activation.

### 3.2. Baicalin Reduces TXNIP-NLRP3 Interactions in Mtb-Infected Macrophages

**Figure 2:**
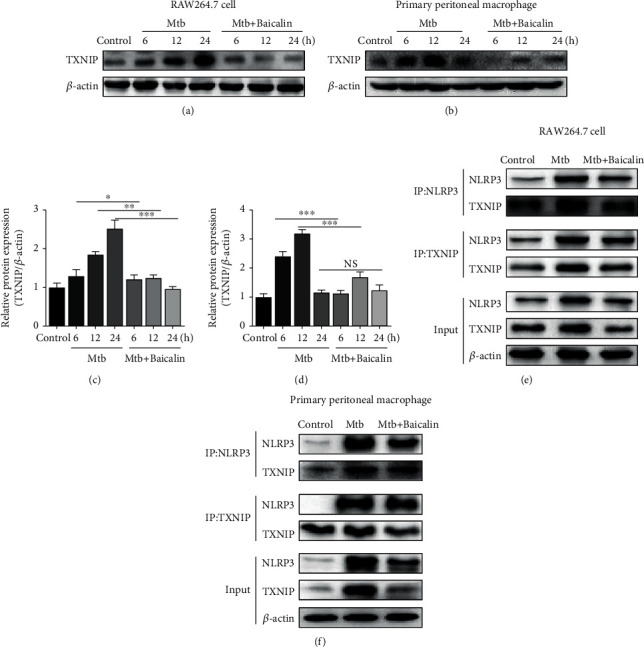
Baicalin reduces TXNIP-NLRP3 interactions in Mtb-infected macrophages. (a, c) Levels of TXNIP protein detected by western blotting analysis in RAW264.7 cells. (b, d) Levels of TXNIP protein determined by western blotting in primary peritoneal macrophage cells. (e, f) TXNIP or NLRP3 immunoprecipitated from RAW264.7 cells and primary peritoneal macrophage cells were immunoblotted for TXNIP or NLRP3, respectively, and reblotted for TXNIP or NLRP3, respectively. Experiment performed three times. ^∗^*p* < 0.05; ^∗∗^*p* < 0.01; ^∗∗∗^*p* < 0.001; NS: nonsignificant.

Generally, the NLRP3 inflammasome can be activated by various pathogen-associated molecular patterns (PAMPs) and damage-associated molecular patterns (DAMPs) [14, 15]. Thioredoxin-interacting protein (TXNIP) is a member of the thioredoxin (TRX) system and an endogenous regulator of redox/glucose-induced stress and inflammation; it is also essential for maintaining the cellular redox status balance and is involved in various disorders including sepsis, traumatic brain injury, diabetes, and Alzheimer's disease (AD) [16–19]. TXNIP upregulates inflammatory responses via decreased expression of TRX, and TRX suppression leads to increased oxidative stress [20]. Inhibiting the dissociation of TRX and TXNIP and blocking the interactions between TXNIP and NLRP3 markedly suppresses NLRP3 inflammasome activation, IL-1*β* maturation, and pyroptosis [21, 22]. As a secondary signal, TXNIP is also a key factor that activates the NLRP3 inflammasome [23]. To examine whether baicalin affects TXNIP protein expression, we measured its expression by western blotting. As shown in Figure 2, Mtb infection increased TXNIP protein levels, whereas baicalin reduced the expression in RAW264.7 cells and primary peritoneal macrophages (Figures 2(a)–2(d)).

**Figure 3:**
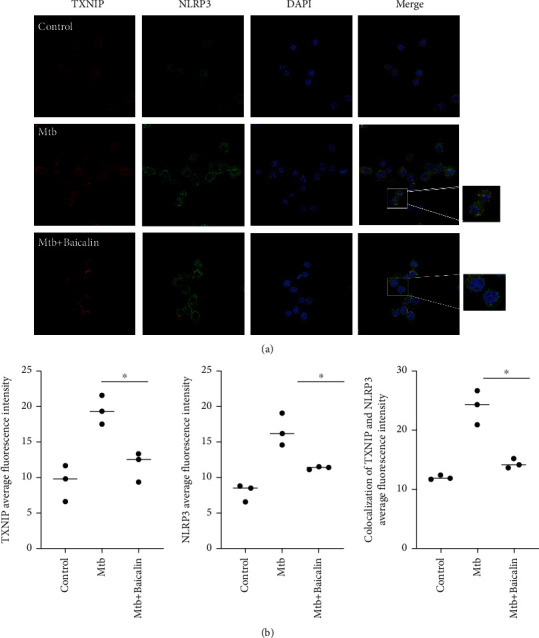
Baicalin prevents the colocalization of TXNIP and NLRP3 in Mtb-infected macrophages. (a, b) Confocal microscopy of RAW264.7 cells with different treatments immunostained with anti-TXNIP (red), anti-NLRP3 (green), and DAPI (blue). Mean ± SD of colocalization of TXNIP and NLRP3, TXNIP and NLRP3 florescence intensity in three independent pictures was shown as scatter plot and error bar (*n* = 3). Kruskal-Wallis *H* rank and inspection was used as the statistical analysis method. According to the statistical results of Kruskal-Wallis *H* rank sum test, *p* < 0.05. The difference between the three groups was statistically significant.

TXNIP binds to NLRP3 through the leucine-rich repeat domain after it dissociates from TRX, and then, the dissociative TXNIP activates the NLRP3 inflammasome in response to oxidative stress via an association with NLRP3 [24]. To assess whether NLRP3 inflammasome activation is due to increased TXNIP expression, coimmunoprecipitation and immunofluorescence were used to analyze the interactions between TXNIP and the NLRP3 inflammasome. These interactions were reduced by baicalin treatment in RAW264.7 cells (Figure 2(e)) and primary peritoneal macrophages (Figure 2(f)). Confocal imaging also showed that Mtb increased the colocalization of TXNIP and NLRP3, and baicalin treatment significantly reduced TXNIP and NLRP3 proteins levels and reduced their colocalization (Figures 3(a) and 3(b)). These results showed that baicalin can significantly reduce the interactions between TXNIP and NLRP3.

### 3.3. Baicalin Reduces TXNIP Expression by Inhibiting ER Stress in Mtb-Infected Macrophages

ER stress plays a key role in inducing NLRP3 inflammasome activation by promoting interactions between TXNIP and NLRP3, thereby increasing the production of various proinflammatory factors and inducing pyroptosis [23, 25, 26]. Recent studies also demonstrated that Mtb infection can induce ER stress [27]. Therefore, we determined whether baicalin could affect TXNIP expression through ER stress in the Mtb-infected model.

**Figure 4:**
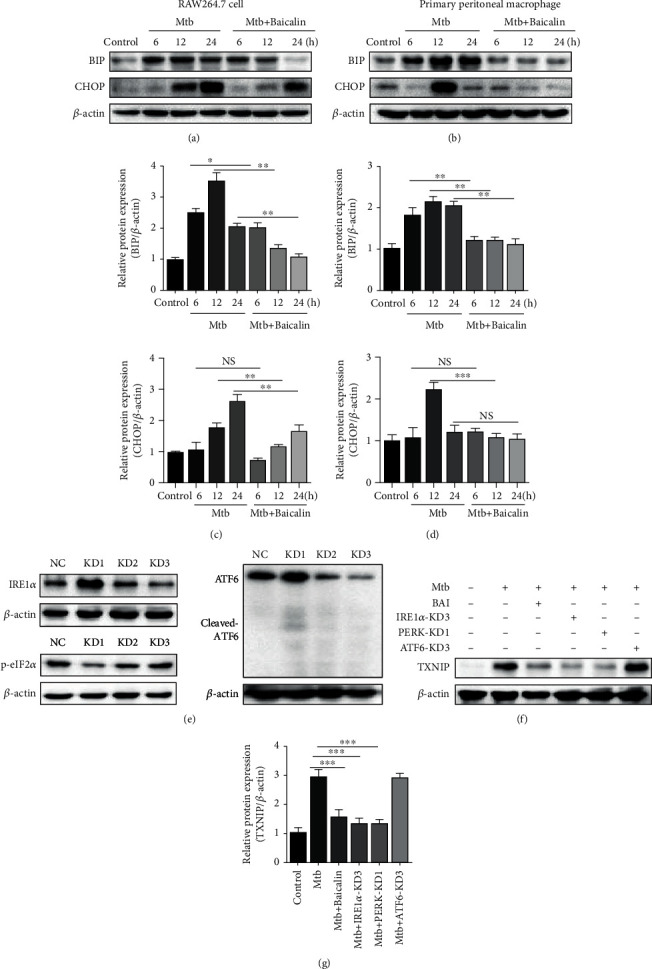
Baicalin attenuates TXNIP via inhibiting endoplasmic reticulum stress in Mtb-infected macrophages. (a, c) Levels of BIP and CHOP protein were tested by western blotting measurement in RAW264.7 cells. (b, d) Levels BIP and CHOP protein were measured by western blotting in primary peritoneal macrophage cells. (e) Transfection efficiency of siRNAs was measured by western blotting analysis in RAW264.7 cells. (f) Western blotting analysis of TXNIP expression after siRNAs of IRE1*α*, PERK, and ATF6, respectively, in RAW264.7 cells. Data are shown as mean ± SD of three independent experiments. ^∗^*p* < 0.05; ^∗∗^*p* < 0.01; ^∗∗∗^*p* < 0.001; NS: nonsignificant.

**Figure 5:**
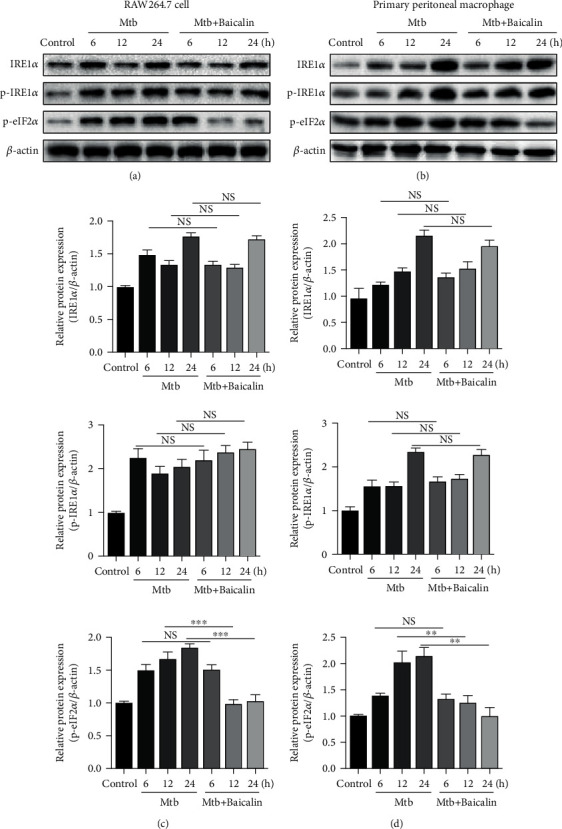
Baicalin inhibits endoplasmic reticulum stress in Mtb-infected macrophages. (a, c) Western blotting analysis of IRE1*α*, p-IRE1*α*, and p-eIF2*α* expression in RAW264.7 cells. (b, d) Levels of IRE1*α*, p-IRE1*α*, and p-eIF2*α* protein assessed by western blotting in primary peritoneal macrophage cells. Data are shown as mean ± SD of at least three independent experiments. ^∗∗^*p* < 0.01; ^∗∗∗^*p* < 0.001; NS: nonsignificant.

Increased BIP and CHOP protein levels are reliable biomarkers of ER stress. Here, we found that ER stress was strongly induced after Mtb infection, whereas baicalin treatment significantly reduced these effects at different time points (Figures 4(a)–4(d)). Moreover, activation of three transmembrane ER-resident stress sensors (IRE1*α*, PERK, and ATF6) is involved in the induction of ER stress. IRE1*α*, PERK, and ATF6 remain inactive after binding to BIP under unstressed conditions. When misfolded proteins accumulate, BIP dissociates from these three ER stress sensors, resulting in the activation of each branch. Previous studies showed that the increase in TXNIP levels depends on the activation of PERK and IRE1*α* [22]. Here, we found that the increase of TXNIP did not depend on the activation of ATF6 by measuring TXNIP protein expression after IRE1*α*, PERK, and ATF6 siRNA transfection (Figures 4(e)–4(g)). These results indicated that baicalin decreased TXNIP protein levels, which depended on the inhibition of IRE1*α* and PERK, rather than the ATF6 pathway. Western blotting was used to assess whether Mtb infection would upregulate IRE1*α* and p-eIF2*α* expression. The results showed that Mtb infection increased PERK/p-eIF2*α* and IRE1*α* expression, whereas baicalin treatment markedly decreased PERK/p-eIF2*α* expression at different time points. However, the increase in IRE1*α* protein levels was not affected by the baicalin treatment (Figures 5(a)–5(d)). These results showed that baicalin reduces TXNIP expression by inhibiting the PERK/eIF2*α* pathway in Mtb-infected macrophages.

### 3.4. Baicalin Reduces Pyroptosis in Mtb-Infected Macrophages by Inhibiting PERK/eIF2*α* Activation

**Figure 6:**
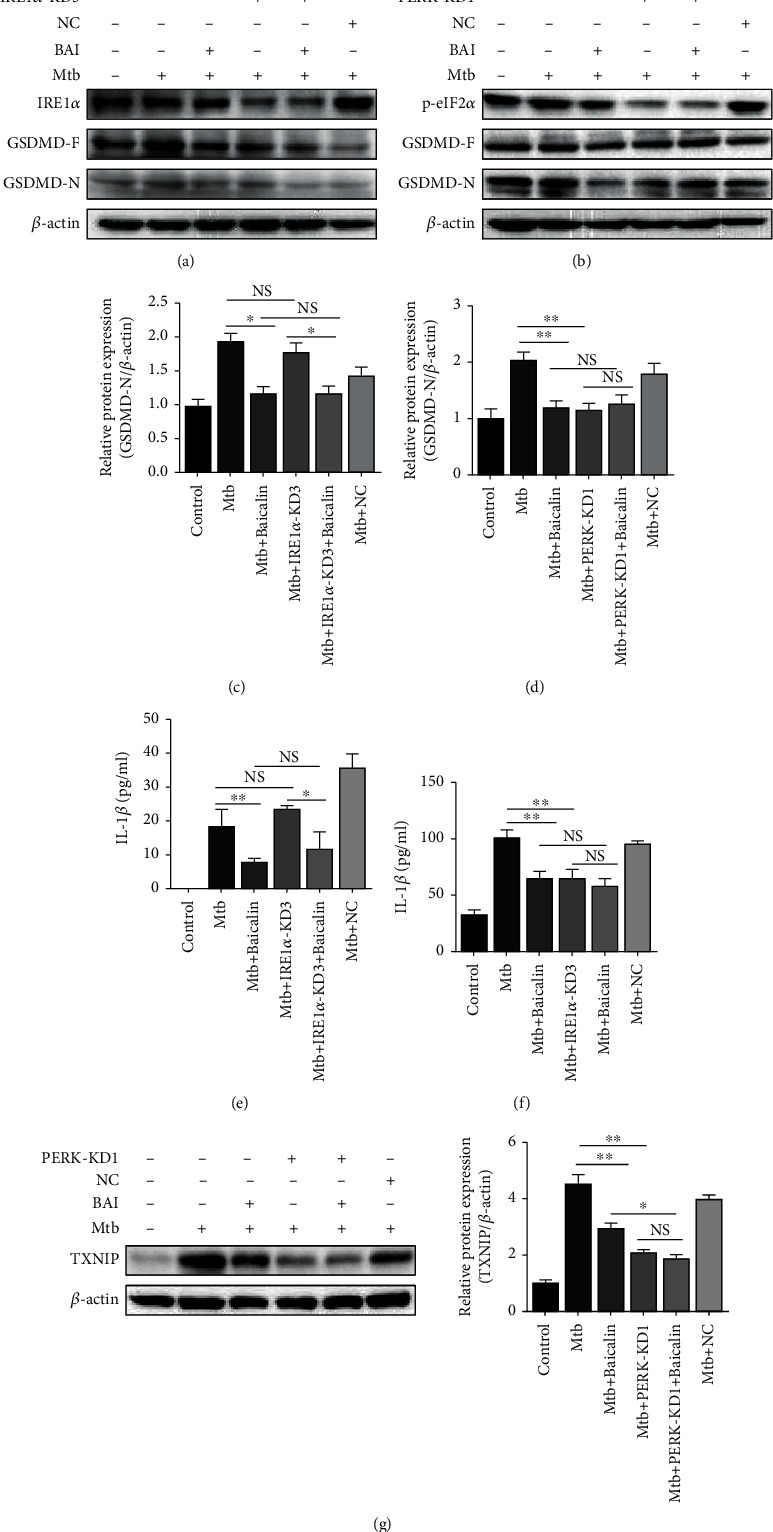
Baicalin reduces pyroptosis in Mtb-infected macrophages by inhibiting PERK/eIF2*α* activation. (a, c) Western blotting analysis of GSDMD-N expression after IRE1*α* siRNA in RAW264.7 cells. (b, d) Level of GSDMD-N protein was detected by western blotting after PERK siRNA in RAW264.7 cells. (e, f) Production of IL-*β* was assessed by ELISA measurement after IRE1*α* or PERK siRNA, respectively, in RAW264.7 cells. (g) Level of TXNIP protein was analyzed by western blotting after PERK siRNA in RAW264.7 cells. Data are shown as mean ± SD of at least three independent experiments. ^∗^*p* < 0.05; ^∗∗^*p* < 0.01; NS: nonsignificant.

A previous study showed that TXNIP expression depends on the activation of the IRE1*α* and PERK pathways, rather than the ATF6 pathway in Mtb-infected macrophages. We determined whether baicalin could inhibit pyroptosis through the IRE1*α* and PERK pathways. Here, we measured the respective effect on pyroptosis by IRE1*α* siRNA and PERK siRNA and assessed GSDMD-N and IL-1*β* levels by western blotting and ELISA. As shown in Figure 6, GSDMD protein expressions in Mtb-infected macrophages did not differ between the IRE1*α* siRNA and non-IRE1*α* siRNA groups (Figures 6(a) and 6(c)). A similar phenomenon was also observed regarding IL-1*β* (Figure 6(e)). These results indicated that pyroptosis is independent on the IRE1*α* pathway in Mtb-infected macrophages.

Similarly, GSDMD protein and IL-1*β* levels were measured after PERK siRNA transfection. GSDMD expression was markedly reduced in Mtb-infected macrophages after PERK siRNA transfection (Figures 6(b) and 6(d)). Similar results were observed for IL-1*β* (Figure 6(f)). Previously, we have revealed that baicalin could suppress the activation of the NLRP3 inflammasome by inhibiting the interaction between TXNIP and the NLRP3 inflammasome. Moreover, the protein levels of TXNIP were decreased after PERK siRNA transfection. To further verify that baicalin could inhibit the activation of the NLRP3 inflammasome by suppressing the activation of the PERK/eIF2*α* pathway in Mtb-infected macrophages, the protein levels of TXNIP were determined by western blotting after PERK siRNA transfection, and TXNIP expression was significantly decreased with this treatment (Figure 6(g)). The results revealed that baicalin can suppress TXNIP expression by inhibiting the activation of the PERK/eIF2*α* pathway. These results demonstrated that pyroptosis depends on the activation of the PERK/eIF2*α* pathway in Mtb-infected macrophages. Taken together, our results demonstrated that Mtb might induce pyroptosis in Mtb-infected macrophages and baicalin can markedly reduce the pyroptosis via inhibition of the PERK/TXNIP/NLRP3 axis.

## 4. Discussion

In the past years, some anti-TB drugs have achieved certain effects. However, due to wide use of these drugs, the drug-resistant Mtb has emerged. Thus, new effective TB drugs are needed [28]. But the research and development of new drugs are very difficult and slow, and thus, there is an urgent need to explore a new method of treating TB.

During active TB, Mtb often induces granuloma and systemic inflammatory responses. Most previous studies predominantly focused on killing Mtb, but the excessive inflammatory response induced by Mtb was ignored. In fact, Mtb could cause excessive inflammatory response to damage tissue and promote the spread of itself. Therefore, reducing excessive inflammation in the host could slow the spread of Mtb and help anti-Mtb drugs work.

HDT is an effective new treatment concept for multidrug-resistant Mtb, and it is aimed at enhancing protective immune responses or reducing exacerbated inflammation, finally to balance host immunity [29]. Based on HDT, we found a new drug for TB—baicalin. It is an active ingredient extracted from Radix Scutellariae and exerts various effects, including anti-inflammatory and antihyperglycemic activities [5–7]. Our previous study showed that baicalin could suppress NLRP3 inflammasome activation by facilitating the degradative mechanism of autophagy [9]. Here, we revealed that baicalin could significantly reduce pyroptosis in Mtb-infected macrophages by inhibiting NLRP3 inflammasome activation.

**Figure 7:**
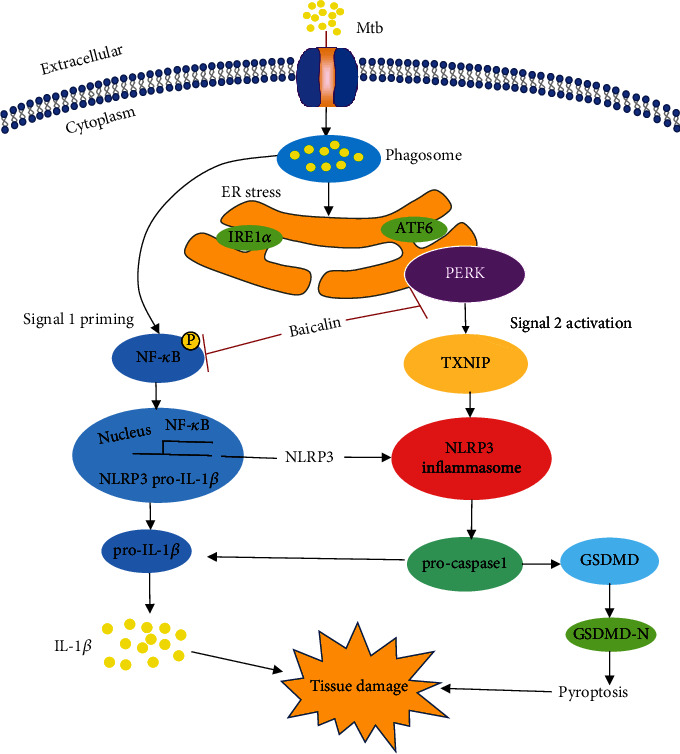
Schematic summary of baicalin inhibiting pyroptosis in Mtb-infected macrophages. Previous study has revealed that baicalin inhibits the NF-*κ*B pathway contributing to the inhibitory effect on NLRP3 inflammasome activation [9]. In the present study, we demonstrated that baicalin also inhibits the activation of the PERK pathway, thus attenuating the production of TXNIP and subsequently alleviating the activation of NLRP3 inflammasome resulting in ameliorating pyroptosis in Mtb-infected macrophages.

Activation of the NLRP3 inflammasome requires two signals, a priming signal and activation signal. The priming signal is induced by microbial components or endogenous cytokines and results in the activation of NF-*κ*B and upregulation of NLRP3, pro-caspase-1, and pro-IL-1*β*. The activation signal is elicited by a series of stimuli including numerous molecular or cellular processes. For example, TXNIP can activate the NLRP3 inflammasome [14, 23]. Previously, we found that baicalin can inhibit the NF-*κ*B pathway, thereby contributing to the inhibitory effect on NLRP3 inflammasome activation [9]. Consistent with those results, here, we revealed that baicalin also inhibits activation of the PERK/eIF2*α* pathway and thus downregulates TXNIP expression and subsequently reduces activation of the NLRP3 inflammasome, resulting in reduced pyroptosis in Mtb-infected macrophages (Figure 7).

Furthermore, recent studies have demonstrated that suppressing the dissociation of TRX and TXNIP and reducing the interactions between TXNIP and NLRP3 significantly inhibited NLRP3 inflammasome activation, IL-1*β* maturation, and pyroptosis [21, 22]. Here, we also revealed that reducing the interactions between TXNIP and NLRP3 with baicalin can contribute to inhibiting the activation of the NLRP3 inflammasome in Mtb-infected macrophages (as shown in Figures 2 and 3). Previous studies demonstrated that baicalin attenuated ER stress induced by tunicamycin through the CHOP/eNOS/NO pathway in cardiomyocytes and prevented lipotoxicity in AML-12 cells by reducing the interactions between TXNIP and the NLRP3 inflammasome [30–33]; here, we also showed this effect.

Moreover, inhibiting the dissociation of TRX and TXNIP and blocking the interactions between TXNIP and NLRP3 markedly suppress NLRP3 inflammasome activation, IL-1*β* maturation, and pyroptosis [21, 22]. The effect of baicalin on inhibiting the interaction between TXNIP and NLRP3 might be due to suppressing the dissociation of TXNIP and TRX, but the specific mechanism through which baicalin inhibits TXNIP and NLRP3 needs to be further explored. Moreover, baicalin can alleviate pyroptosis in hepatocytes by inhibiting NLRP3-GSDMD signaling in vitro and reduces age-related macular degeneration through NLRP3-regulated pyroptosis [33, 34]. However, the current study showed that baicalin ameliorated pyroptosis in Mtb-infected macrophages by inhibiting the interactions between TXNIP and NLRP3.

ER stress plays a key role in human diseases, including colitis and acute pancreatitis, by increasing the release of proinflammatory factors such as IL-1*β*, cleaved caspase-1, and TNF-*α* which is elicited by TXNIP during neuroinflammation. Other studies demonstrated that ER stress can result in NLRP3 inflammasome activation, and Mtb stimulates consistent IL-1*β* production and pyroptosis. When ER stress occurs, three transmembrane proteins (protein kinase-like ER kinase (PERK), activating transcription factor 6 (ATF6), and inositol-requiring enzyme 1*α* (IRE1*α*)) are separated from the chaperone binding immunoglobulin protein (BIP) in response to unfolded or misfolded proteins [34]. Once activated, PERK phosphorylates eukaryotic initiation factor 2*α* (eIF-2*α*), which would attenuate translational initiation and block new proteins in the ER [35]. Severe ER stress induces the activation of the CCAAT-enhancer-binding protein homologous protein (CHOP) [36]. Furthermore, upregulation of IRE1*α* can elicit dissociation of TRX and TXNIP, after which TXNIP binds to the NLRP3 inflammasome and activates it [37, 38]. Moreover, ER stress can increase TXNIP expression through activation of the PERK or IRE1*α* signaling pathway [39–41]. Consistent with these results, our study showed that baicalin suppressed the expression of PERK/p-eIF2*α*. Moreover, we also found that the upregulation of TXNIP was achieved by activation of the IRE1*α* and PERK pathways. However, p-eIF2*α* protein expression was reduced by baicalin at different time points, whereas the expression of IRE1*α* and p-IRE1*α* in Mtb-infected macrophages was not affected. Our results also showed that GSDMD-N protein expression decreased after PERK siRNA transfection, but there was no change after IRE1*α* siRNA transfection (Figure 5). This indicated that baicalin reduced pyroptosis by suppressing the activation of the PERK/p-eIF2*α* pathway. The results of IL-1*β* were similar to those of GSDMD-N. Thus, we proposed that baicalin reduced pyroptosis by inhibiting the PERK/TXNIP/NLRP3 axis. To prove that the effect of baicalin on pyroptosis is extensive and associated with stabilization, we chose two cell lines, RAW264.7 cells and primary peritoneal macrophage cells. These two cell lines are both macrophage cells; RAW264.7 is the most commonly used cell line to study the role of macrophages in bacterial infections and inflammatory responses, whereas primary peritoneal macrophage cells are more sensitive to the stimulation by bacterial infection. All of our results were confirmed in both cell lines.

In conclusion, baicalin markedly reduced pyroptosis in Mtb-infected macrophages, which suggested that it is a novel candidate adjuvant drug for HDT to anti-TB. Additionally, our study generated new research insights regarding TB that might help to identify more effective and specific HDT targets. Drugs such as baicalin can alter host cellular defense mechanisms and prevent host tissue damage during Mtb infection. Taken together, baicalin showed promising effects to prevent pyroptosis in Mtb-infected macrophages through the inhibition of the PERK/TXNIP/NLRP3 axis.

In this study, we used Mtb strain H37Ra as a source of infection. It is an attenuated strain isolated from the standard virulent strain H37Rv, to which it is very similar genetically. Because H37Ra is less virulent, more simple to use, and safer, we used RAW264.7 cells and primary peritoneal macrophages infected with H37Ra as an infection model system [42]. Further research would be necessary to examine the effect of baicalin in combination with anti-TB drugs on animal models of H37Rv infection.

## Data Availability

The data used to support the findings of this study are available from the corresponding author upon request.

## Supplementary Materials

Supplementary Materials Figure S1: the chemical structure of baicalin. Figure S2: baicalin evidently inhibits the Mtb-induced pyroptosis. Figure S3: baicalin attenuates pyroptosis in Mtb-infected macrophages.

## References

[1] Zumla A., Rao M., Parida S. K. (2015). Inflammation and tuberculosis: host-directed therapies. *Journal of Internal Medicine*.

[2] Schito M., Migliori G. B., Fletcher H. A. (2015). Perspectives on advances in tuberculosis diagnostics, drugs, and vaccines. *Clinical infectious diseases*.

[3] Frank D. J., Horne D. J., Dutta N. K. (2019). Remembering the host in tuberculosis drug development. *The Journal of infectious diseases*.

[4] Tiberi S., du Plessis N., Walzl G. (2018). Tuberculosis: progress and advances in development of new drugs, treatment regimens, and host-directed therapies. *The Lancet Infectious Diseases*.

[5] Guo L. T., Wang S. Q., Su J. (2019). Baicalin ameliorates neuroinflammation-induced depressive-like behavior through inhibition of toll-like receptor 4 expression via the PI3K/AKT/FoxO1 pathway. *Journal of Neuroinflammation*.

[6] Ding L., Jia C., Zhang Y. (2019). Baicalin relaxes vascular smooth muscle and lowers blood pressure in spontaneously hypertensive rats. *Biomedicine & Pharmacotherapy*.

[7] Xu J., Li Y., Lou M. (2018). Baicalin regulates SirT1/STAT3 pathway and restrains excessive hepatic glucose production. *Pharmacological Research*.

[8] Lin M., Li L., Zhang Y. (2014). Baicalin ameliorates H2O2 induced cytotoxicity in HK-2 cells through the inhibition of ER stress and the activation of Nrf2 signaling. *International Journal of Molecular Sciences*.

[9] Zhang Q., Sun J., Wang Y. (2017). Antimycobacterial and anti-inflammatory mechanisms of baicalin via induced autophagy in macrophages infected with Mycobacterium tuberculosis. *Frontiers in Microbiology*.

[10] Jiang X., Wang Y., Qin Y. (2017). Micheliolide provides protection of mice against *Staphylococcus aureus* and MRSA infection by down-regulating inflammatory response. *Scientific Reports*.

[11] Qu Z., Zhou J., Zhou Y. (2020). Mycobacterial EST12 activates a RACK1-NLRP3-gasdermin D pyroptosis-IL-1*β* immune pathway. *Science Advances*.

[12] Beckwith K. S., Beckwith M. S., Ullmann S. (2020). Plasma membrane damage causes NLRP3 activation and pyroptosis during *Mycobacterium tuberculosis* infection. *Nature Communications*.

[13] Wang Y. A.-O., Zhang H., Chen Q. (2020). TNF-*α*/HMGB1 inflammation signalling pathway regulates pyroptosis during liver failure and acute kidney injury. *Cell Proliferation*.

[14] Zhou R., Yazdi A. S., Menu P., Tschopp J. (2011). A role for mitochondria in NLRP3 inflammasome activation. *Nature*.

[15] Elliott E. I., Sutterwala F. S. (2015). Initiation and perpetuation of NLRP3 inflammasome activation and assembly. *Immunological Reviews*.

[16] Yang Y., Wang H., Kouadir M., Song H., Shi F. (2019). Recent advances in the mechanisms of NLRP3 inflammasome activation and its inhibitors. *Cell Death & Disease*.

[17] Mahmood D. F., Abderrazak A., el Hadri K., Simmet T., Rouis M. (2013). The thioredoxin system as a therapeutic target in human health and disease. *Antioxidants & Redox signaling*.

[18] Ma M. A.-O., Wang J., Dhandapani K. M., Brann D. W. (2017). NADPH oxidase 2 regulates NLRP3 inflammasome activation in the brain after traumatic brain injury. *Oxidative medicine and cellular longevity*.

[19] Szpigel A., Hainault I., Carlier A. (2018). Lipid environment induces ER stress, TXNIP expression and inflammation in immune cells of individuals with type 2 diabetes. *Diabetologia*.

[20] Tseng P. C., Kuo C. F., Cheng M. H. (2019). HECT E3 ubiquitin ligase-regulated Txnip degradation facilitates TLR2-mediated inflammation during group A streptococcal infection. *Frontiers in Immunology*.

[21] Han Y., Xu X., Tang C. (2018). Reactive oxygen species promote tubular injury in diabetic nephropathy: the role of the mitochondrial ros-txnip-nlrp3 biological axis. *Redox Biology*.

[22] Jia Y., Cui R., Wang C. (2020). Metformin protects against intestinal ischemia-reperfusion injury and cell pyroptosis via TXNIP-NLRP3-GSDMD pathway. *Redox Biology*.

[23] Li L., Ismael S., Nasoohi S. (2019). Thioredoxin-interacting protein (TXNIP) associated NLRP3 inflammasome activation in human Alzheimer’s disease brain.. *Journal of Alzheimer's Disease*.

[24] Zhou R., Tardivel A., Thorens B., Choi I., Tschopp J. (2010). Thioredoxin-interacting protein links oxidative stress to inflammasome activation. *Nature Immunology*.

[25] Sprenkle N. T., Sims S. G., Sánchez C. L., Meares G. P. (2017). Endoplasmic reticulum stress and inflammation in the central nervous system.. *Molecular Neurodegeneration*.

[26] Lerner A. G., Upton J. P., Praveen P. V. K. (2012). IRE1*α* induces thioredoxin-interacting protein to activate the NLRP3 inflammasome and promote programmed cell death under irremediable ER stress. *Cell Metabolism*.

[27] Kim S. H., Cho S. N., Lim Y. J. (2018). Phagocytosis influences the intracellular survival of Mycobacterium smegmatis via the endoplasmic reticulum stress response. *Cell & Bioscience*.

[28] Seung K. J., Keshavjee S., Rich M. L. (2015). Multidrug-resistant tuberculosis and extensively drug-resistant tuberculosis. *Cold Spring Harbor Perspectives in Medicine*.

[29] Kaufmann S. H. E., Dorhoi A., Hotchkiss R. S., Bartenschlager R. (2018). Host-directed therapies for bacterial and viral infections. *Nature reviews Drug discovery*.

[30] Shen M., Wang L., Yang G. (2014). Baicalin protects the cardiomyocytes from ER stress-induced apoptosis: inhibition of CHOP through induction of endothelial nitric oxide synthase. *PLoS One*.

[31] Zhang J., Zhang H., Deng X., Zhang Y., Xu K. (2017). Baicalin protects AML-12 cells from lipotoxicity via the suppression of ER stress and TXNIP/NLRP3 inflammasome activation. *Chemico-Biological Interactions*.

[32] Shi H., Zhang Y., Xing J. (2020). Baicalin attenuates hepatic injury in non-alcoholic steatohepatitis cell model by suppressing inflammasome-dependent GSDMD-mediated cell pyroptosis. *International Immunopharmacology*.

[33] Sun H. J., Jin X. M., Xu J., Xiao Q. (2020). Baicalin alleviates age-related macular degeneration via miR-223/NLRP3-regulated pyroptosis. *Pharmacology*.

[34] Latham K. E. (2016). Stress signaling in mammalian oocytes and embryos: a basis for intervention and improvement of outcomes. *Cell and Tissue Research*.

[35] Teske B. F., Wek S. A., Bunpo P. (2011). The eIF2 kinase PERK and the integrated stress response facilitate activation of ATF6 during endoplasmic reticulum stress. *Molecular Biology of the Cell*.

[36] Yang Y., Pei X., Jin Y., Wang Y., Zhang C. (2016). The roles of endoplasmic reticulum stress response in female mammalian reproduction. *Cell and Tissue Research*.

[37] Ke R., Wang Y., Hong S., Xiao L. (2020). Endoplasmic reticulum stress related factor IRE1*α* regulates TXNIP/NLRP3-mediated pyroptosis in diabetic nephropathy. *Experimental Cell Research*.

[38] Yang Y., Li J., Han T. L. (2020). Endoplasmic reticulum stress may activate NLRP3 inflammasomes via TXNIP in preeclampsia. *Cell and Tissue research*.

[39] Powell N. A.-O., Pantazi E., Pavlidis P. (2020). Interleukin-22 orchestrates a pathological endoplasmic reticulum stress response transcriptional programme in colonic epithelial cells. *Gut*.

[40] Lee P. J., Papachristou G. I. (2019). New insights into acute pancreatitis. *Nature Reviews Gastroenterology & Hepatology*.

[41] Xu W., Li T., Gao L. (2019). Apelin-13/APJ system attenuates early brain injury via suppression of endoplasmic reticulum stress-associated TXNIP/NLRP3 inflammasome activation and oxidative stress in a AMPK-dependent manner after subarachnoid hemorrhage in rats. *Journal of neuroinflammation*.

[42] Zheng H., Lu L., Wang B. (2008). Genetic basis of virulence attenuation revealed by comparative genomic analysis of Mycobacterium tuberculosis strain H37Ra versus H37Rv. *PLoS one*.

