# Supplementary material for: Inhibition of the PERK/TXNIP/NLRP3 Axis by Baicalin Reduces NLRP3 Inflammasome-Mediated Pyroptosis in Macrophages Infected with Mycobacterium tuberculosis
Source: Mediators Inflamm. 2021 Nov 8;2021:1805147. doi: 10.1155/2021/1805147 (PMC8592748; doi:10.1155/2021/1805147)
Supplement: Supplementary Materials — Figure S1: the chemical structure of baicalin. Figure S2: baicalin evidently inhibits the Mtb-induced pyroptosis. Figure S3: baicalin attenuates pyroptosis in Mtb-infected macrophages. [file 1805147.f1.doc]

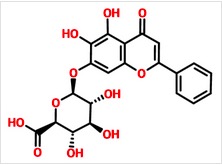


**Figure S1.** The chemical structure of baicalin.

**
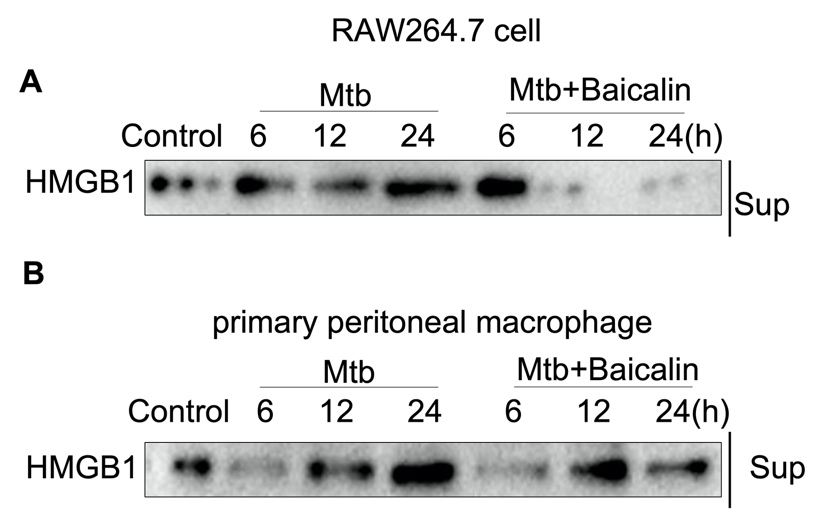
**

**Figure S2.** Baicalin evidently inhibits the Mtb-induced pyroptosis.

**A, B:** Levels of HMGB1 in the supernatant was analyzed by western blotting in RAW264.7 cell and primary peritoneal macrophage respectively.

**
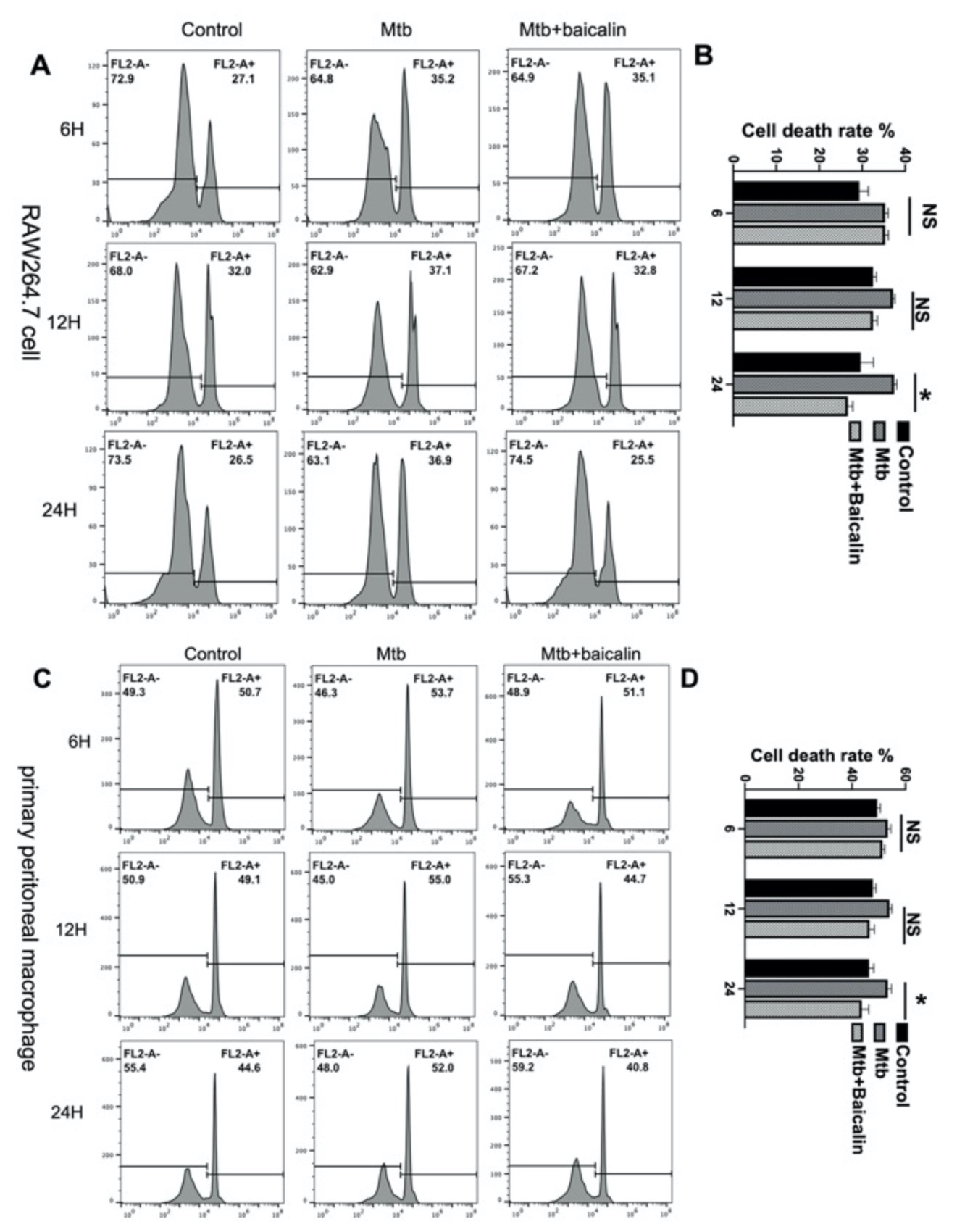
**

**Figure S3.** Baicalin attenuates pyroptosis in Mtb-infected macrophages. **(A, B)** The viable and dead cell populations were measured by PI staining in RAW264.7 cells. **(C, D)** The viable and dead cell populations were measured by PI staining in primary peritoneal macrophage cells. FL2-A+ represents the dead cell populations, FL2-A- represents the viable cell populations. Data are shown as mean ± SD of three independent experiments.
